# Brain-specific glycosylation enzyme GnT-IX maintains levels of protein tyrosine phosphatase receptor PTPRZ, thereby mediating glioma growth

**DOI:** 10.1016/j.jbc.2023.105128

**Published:** 2023-08-04

**Authors:** Kenichiro Nagai, Yui Muto, Saori Miura, Kazuto Takahashi, Yu Naruse, Ryo Hiruta, Yuko Hashimoto, Miwa Uzuki, Yoshimi Haga, Risa Fujii, Koji Ueda, Yasushi Kawaguchi, Masazumi Fujii, Shinobu Kitazume

**Affiliations:** 1 Department of Neurosurgery, Fukushima Medical University, Fukushima, Japan; 2 Division of Molecular Virology, Department of Microbiology and Immunology, The Institute of Medical Science, The University of Tokyo, Tokyo, Japan; 3 Department of Clinical Laboratory Sciences, School of Health Sciences, Fukushima Medical University, Fukushima, Japan; 4 Department of Diagnostic Pathology, Fukushima Medical University, Fukushima, Japan; 5 Cancer Proteomics Group, Cancer Precision Medicine Center, Japanese Foundation for Cancer Research, Tokyo, Japan; 6 Department of Infectious Disease Control, International Research Center for Infectious Diseases, The Institute of Medical Science, The University of Tokyo, Tokyo, Japan; 7 Research Center for Asian Infectious Diseases, The Institute of Medical Science, The University of Tokyo, Tokyo, Japan

**Keywords:** PTPRZ, HNK-1, O-Man glycan, GnT-IX, glycosylation, glioma, therapeutic target

## Abstract

Gliomas are the most prevalent primary tumor of the central nervous system. Despite advances in imaging technologies, neurosurgical techniques, and radiotherapy, a cure for high-grade glioma remains elusive. Several groups have reported that protein tyrosine phosphatase receptor type Z (PTPRZ) is highly expressed in glioblastoma, and that targeting PTPRZ attenuates tumor growth in mice. PTPRZ is modified with diverse glycan, including the PTPRZ-unique human natural killer-1 capped O-mannosyl core M2 glycans. However, the regulation and function of these unique glycans are unclear. Using CRISPR genome-editing technology, we first demonstrated that disruption of the PTPRZ gene in human glioma LN-229 cells resulted in profoundly reduced tumor growth in xenografted mice, confirming the potential of PTPRZ as a therapeutic target for glioma. Furthermore, multiple glycan analyses revealed that PTPRZ derived from glioma patients and from xenografted glioma expressed abundant levels of human natural killer-1–capped O-Man glycans *via* extrinsic signals. Finally, since deficiency of O-Man core M2 branching enzyme *N*-acetylglucosaminyltransferase IX (GnT-IX) was reported to reduce PTPRZ protein levels, we disrupted the GnT-IX gene in LN-229 cells and found a significant reduction of glioma growth both *in vitro* and in the xenograft model. These results suggest that the PTPR glycosylation enzyme GnT-IX may represent a promising therapeutic target for glioma.

Gliomas are the most common primary tumors in the central nervous system (CNS) (1, 2). High-grade gliomas—such as oligodendroglioma, isocitrate dehydrogenase (IDH) mutant, and 1p/19q-codeleted, World Health Organization (WHO) grade 3; astrocytoma, IDH mutant, WHO grade 3/4; and glioblastoma, IDH wildtype, WHO grade 4—exhibit highly invasive and proliferative phenotypes. Despite advances in surgery, radiation, and chemotherapy treatments, the median survival of patients with glioblastoma, the highest-grade glioma, is only 15 months (3).

Protein tyrosine phosphatase receptor type Z (PTPRZ) is a membrane protein that is abundantly expressed in CNS glial cells (4), including oligodendrocyte precursor cells, astrocytes, and oligodendrocytes (5). Although the multiregulatory roles of PTPRZ—such as in the control of oligodendrocyte precursor cell development (6), regulation of the remyelination process (7, 8), and the formation of perineuronal nets (9)—are well discussed, PTPRZ-deficient mice exhibit no obvious abnormalities (10). PTPRZ is abundant in gliomas (11, 12, 13), and its soluble cleaved form (sPTPRZ) is detected at high concentrations in the cerebrospinal fluid (CSF) of glioma patients, indicating that CSF sPTPRZ might be a diagnostic marker for glioma (14). Furthermore, several reports have suggested that PTPRZ-dependent signaling *via* its ligand pleiotrophin, which is abundantly secreted from tumor-associated macrophages (15), neural precursor cells (16), and glioma cells (17), supports glioma growth and invasion and the maintenance of glioma stem cells (15). These findings indicate that targeting PTPRZ may be a promising glioma therapy. Indeed, both small interfering RNA targeting PTPRZ and small-molecule PTPRZ inhibitors significantly reduce tumor growth *in vivo* (18, 19, 20). However, the relatively large catalytic pocket of PTPRZ (19) has hampered the development of small-molecule inhibitors that can cross the blood–brain barrier.

Brain PTPRZ undergoes several types of glycosylation (21, 22), such as by chondroitin sulfate (23), keratan sulfate (24), N-glycans, GalNAc-type O-glycans, and O-mannosyl (O-Man) glycans. Notably, several types of PTPRZ glycans are brain specific. The brain-specific human natural killer-1 (HNK-1) epitope is attached to the nonreducing ends of both N-glycans and O-Man core M1 and M2 glycans of PTPRZ (22, 25, 26, 27). Formation of the core M2 branch structure is initiated by the brain-specific *N*-acetylglucosaminyltransferase IX (GnT-IX) (28, 29). To date, HNK-1-capped O-Man core M2 glycans have only been identified in PTPRZ. Here, we used CRISPR genome-editing technology and demonstrated that disruption of the PTPRZ gene in human glioma LN-229 cells resulted in profoundly reduced tumor growth in xenografted mice, suggesting that PTPRZ may be a potential therapeutic target for glioma. Because an important role of protein glycosylation is to protect proteins (30) and a previous study has reported that GnT-IX-deficient mice have reduced PTPRZ in the brain (29), we also explored whether disruption of the GnT-IX gene results in reduced glioma growth. Indeed, GnT-IX knockdown in glioma cells led to reduced cellular PTPRZ and a marked decrease in xenograft tumor growth. Our data demonstrate that, similar to PTPRZ, GnT-IX is a promising target for glioma therapies.

## Results

### PTPRZ deficiency reduces glioma growth

**Figure 1:**
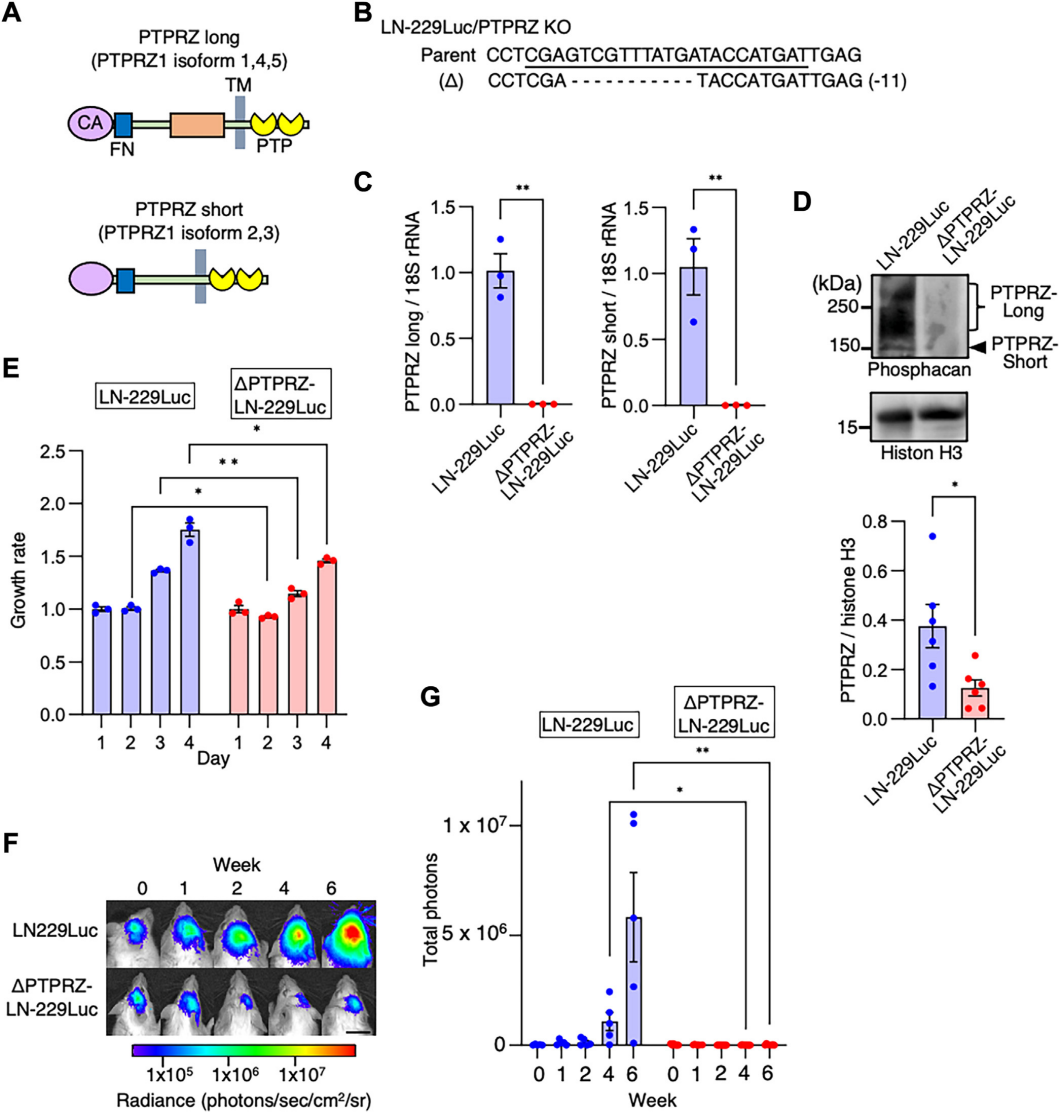
**PTPRZ depletion attenuates glioma growth both *in vitro* and *in vivo*.***A*, schematic of two types of PTPRZ, produced by alternative mRNA splicing. Compared with PTPRZ-long, PTPRZ-short lacks a juxtamembrane extracellular region (∼900 amino acids). *B*, the targeted PTPRZ mutation sequences in ΔPTPRZ-LN-229Luc cells and the parental sequence are shown. The single-guide RNA target sequence is *underlined*. *C*, quantification of PTPRZ-long and PTPRZ-short mRNA transcripts, normalized to ribosomal RNA, in LN-229Luc and ΔPTPRZ-LN-229Luc cells. Data are shown as the mean ± SEM (n = 3). Student’s *t* test; ∗∗*p* < 0.01. *D*, immunoblot analysis of PTPRZ with histone H3 as the loading control in LN-229Luc and ΔPTPRZ-LN-229Luc cells. Data in the graph are shown as the mean ± SEM (n = 5). Student’s *t* test; ∗*p* < 0.05. *E*, cell growth rates of LN-229Luc and ΔPTPRZ-LN-229Luc cells with day 1 set as 1. Data are shown as the mean ± SEM (n = 3). Student’s *t* test; ∗*p* < 0.05 and ∗∗*p* < 0.01. *F*, *in vivo* bioluminescent analysis to monitor the intracranial tumor growth of glioblastoma xenografts derived from LN-229Luc and ΔPTPRZ-LN-229Luc cells in mice. Representative bioluminescent images on the indicated days are shown. Scale bar represents 1 cm. *G*, mean bioluminescence of glioblastoma xenografts with LN-229Luc and ΔPTPRZ-LN-229Luc from 0 to 6 weeks. Data are shown as the mean ± SEM. n = 5 mice per group (LN-229Luc-derived xenografts) or n = 7 mice per group (ΔPTPRZ-LN-229Luc-derived xenografts). Student’s *t* test; ∗*p* < 0.05 and ∗∗*p* < 0.01. CA, carbonic anhydrase; FN, fibronectin type III; PTP, protein tyrosine phosphatase; PTPRZ, protein tyrosine phosphatase receptor Z; TM, transmembrane.

Using antibodies, small-molecule inhibitors, or small hairpin RNA in glioma tumors in mouse xenograft models, several groups have reported that PTPRZ is a promising therapeutic target for glioma (19, 20, 31). To confirm this, we used CRISPR genome-editing technology and disrupted the PTPRZ gene in LN-229Luc cells (human glioma LN-229 cells that stably express luciferase and GFP). As a result of alternative mRNA splicing, PTPRZ has multiple mRNA isoforms; in humans, there are two main groups: PTPRZ-long and PTPRZ-short (Fig. 1*A*) (32). Quantitative PCR analysis revealed that LN-229 expressed both PTPRZ isoforms, whereas levels of both types of PTPRZ expression were negligible in the PTPRZ knockdown clone Δ-PTPRZ-LN-229Luc (Fig. 1, *B* and *C*). Western blot analysis revealed that PTPRZ-long and PTPRZ-short levels were decreased in ΔPTPRZ-LN-229Luc compared with LN-229Luc (Fig. 1*D*). Furthermore, *in vitro* cell proliferation assays demonstrated that cell growth rates were significantly lower in ΔPTPRZ-LN-229Luc than in LN-229-Luc from day 2 of incubation (Fig. 1*E*). We also investigated whether PTPRZ knockdown suppressed tumor growth in a xenograft glioma model. LN-229Luc or ΔPTPRZ-LN-229Luc were transplanted into the brains of SCID-Beige mice, which are defective in T- and B-cell development and natural killer cell activity; tumor growth was monitored every week using an *in vivo* imaging system. Although LN-229Luc-transplanted mice showed tumor growth over time, tumor growth was significantly suppressed with ΔPTPRZ-LN-229Luc transplantation; marked differences were observed from week 4 after transplantation (Fig. 1*F*). Similar to previous reports (19, 20, 31), our results indicate the important role of PTPRZ in glioma growth.

### Glycosylation of PTPRZ in glioma

**Figure 2:**
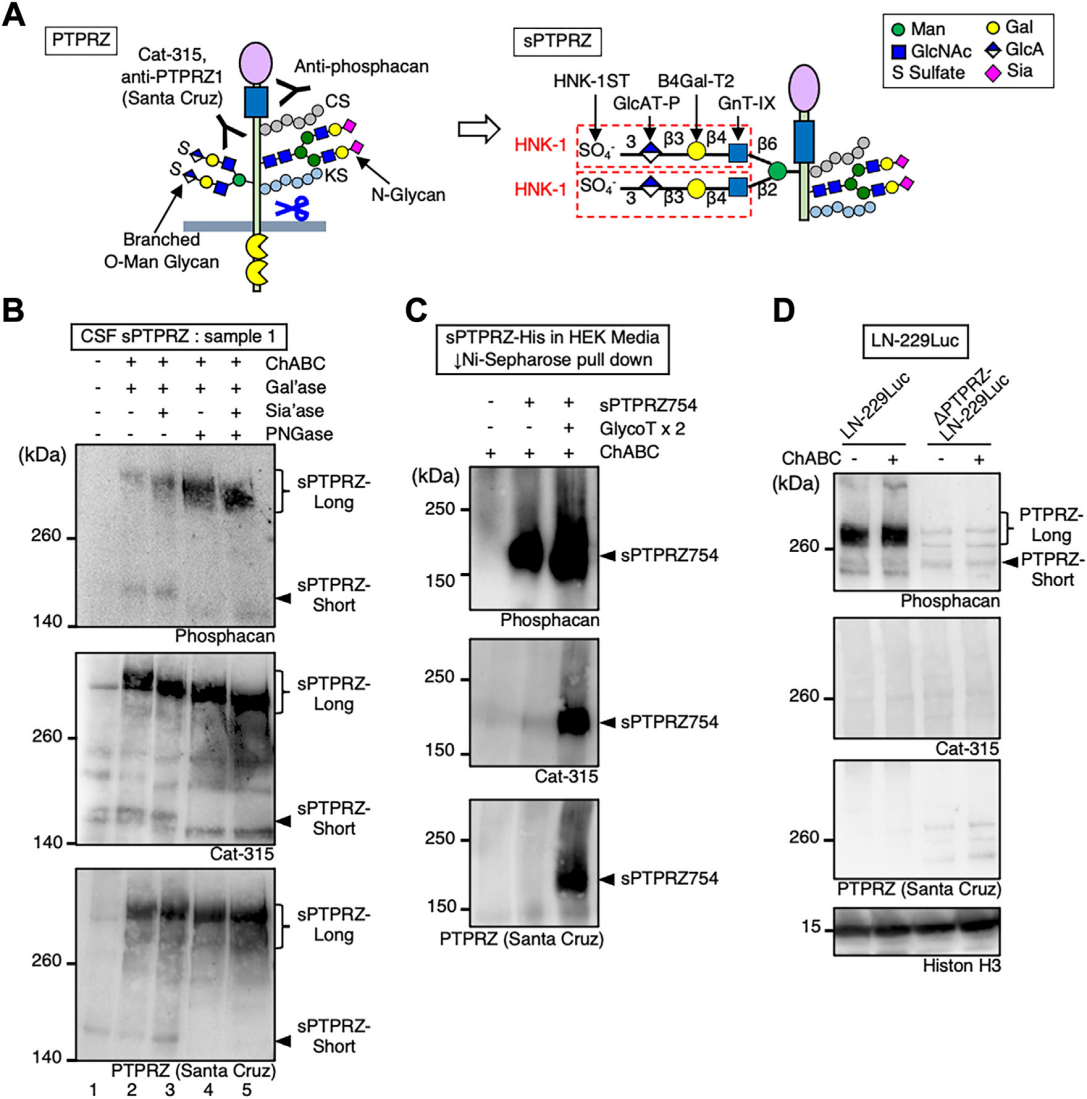
**The soluble cleaved form of PTPRZ (sPTPRZ) in cerebrospinal fluid (CSF) is highly glycosylated.***A*, schematic structures of PTPRZ and sPTPRZ, modified with representative glycans. HNK-1-capped O-Man core M2 glycan and a series of glycosyltransferases required for its glycan synthesis are shown. *GnT-IX*, *β1,6-N-acetylglucosaminyltransferase-5b*; *B4Gal-T2*, *β1,4-galactosyltransferase 2*; *GlcAT-P, β1,3-glucuronyl transferase 1*; *and HNK-1ST, carbohydrate sulfotransferase 10*. *B*, immunoblot analysis of sPTPRZ in the CSF of a glioma patient. CSF samples from a glioma patient were treated with or without chondroitinase ABC (ChABC), endo-β-galactosidase (Gal’ase), sialidase (Sia’ase), and peptide-*N*-glycosidase (PNGase) and then probed with a series of antibodies: antiphosphacan, anti-PTPRZ (Santa Cruz), and Cat-315. *C*, sPTPRZ754-His in the culture media of HEK293T with or without GlcAT-P plus HNK-1ST (GLycoT X2 cells were pulled down with Ni-Sepharose, treated with or without ChABC and used for Western blot analysis). *D*, cell lysates of LN-229Luc and ΔPTPRZ-LN-229Luc were treated with or without ChABC and used for Western blot analysis. HEK293T, human embryonic kidney 293T cell line; HNK-1, human natural killer-1; PTPRZ, protein tyrosine phosphatase receptor Z.

The extracellular region of PTPRZ is heavily glycosylated, cleaved, and shed (33), and the resulting soluble form is known as sPTPRZ or phosphacan (Fig. 2*A*). We have previously reported that sPTPRZ is detectable in CSF; its level is ten times higher in patients with glioma than in those with other brain diseases such as multiple sclerosis (14). To explore the glycosylation status of PTPRZ in glioma, we treated sPTPRZ in CSF from glioma patients with several kinds of glycosidases: chondroitinase ABC (ChABC), end-β-galactosidase, sialidase, and peptide-*N*-glycosidase (PNGase). We used three different antibodies to detect PTPRZ: Cat-315, anti-PTPRZ (Santa Cruz), and antiphosphacan. Antiphosphacan is raised against recombinant phosphacan (sPTPRZ) (34), whereas Cat-315 detects the HNK-1-capped O-Man glycan plus PTPRZ peptide region (35, 36). The epitope information of anti-PTPRZ (Santa Cruz) has not yet been investigated in detail, but both Cat-315 and anti-PTPRZ (Santa Cruz) antibodies react with sPTPRZ-long and sPTPRZ-short in CSF (14). After ChABC and end-β-galactosidase digestions to remove chondroitin sulfate and keratan sulfate, a sPTPRZ-long signal (300–500 kDa) was detected with antiphosphacan, Cat-315, and anti-PTPRZ (Santa Cruz), indicating that the domain specific to PTPRZ-long is modified with these glycosaminoglycan chains to mask epitope regions (lanes 1 and 2, Figs. 2*B* and S1), as reported previously (23). These antibodies also detected sPTPRZ-short (∼200 kDa). Sialidase digestion reduced the molecular weights of sPTPRZ-long and sPTPRZ-short, indicating that both forms are sialylated (lanes 2 and 3). After PNGase digestion to remove N-glycans, the bands corresponding to sPTPRZ-long and sPTPRZ-short were shifted, indicating that both forms have N-glycans (lanes 2 and 4) (35, 36). Sialic acid was also present on O-glycan because sialidase digestion reduced the molecular weight of sPTPRZ-long without N-glycans (lanes 4 and 5). Compared with Cat-315 and anti-PTPRZ (SantaCruz), antiphosphacan reacted weakly with sPTPRZ even after the removal of glycosaminoglycan chains; however, additional glycosidase treatment enhanced the sPTPRZ signals, indicating that glycosylation hinders the epitope of the antiphosphacan antibody.

### Glycosylation analysis of PTPRZ in cell culture

Because both Cat-315 and anti-PTPRZ (Santa Cruz) clearly detected CSF sPTPRZ and their Western blot patterns of glycosidase-treated samples were similar, we speculated that both antibodies recognize brain-specific HNK-1 epitopes on PTPRZ, and that this epitope is crucial for detecting CSF sPTPRZ. To test this idea, we expressed human PTPRZ in human embryonic kidney 293T (HEK293T) cells with or without two types of glycosyltransferases, GlcAT-P (pIRES-glucuronyltransferase) and HNK-1 sulfotransferase (HNK-1ST) (GlycoT X2), which are key enzymes for HNK-1 epitope synthesis. We first expressed full-length PTPRZ-long; however, sPTPRZ-long showed high instability and was almost undetectable in culture media, as has been previously reported (37). We then expressed a shorter sPTPRZ, His-tagged-sPTPRZ754 (sPTPRZ754-His), which lacks transmembrane and cytoplasmic regions and has a His tag at the C terminus. Without GlycoT X2 expression, sPTPRZ754-His was detected using antiphosphacan but not Cat-315 or anti-PTPRZ (Santa Cruz) (lane 2, Fig. 2*C*). In contrast, with GlycoT X2 expression, sPTPRZ754-His was clearly detected not only with antiphosphacan but also with Cat-315 and anti-PTPRZ (Santa Cruz) (lane 3). These findings indicate that both Cat-315 and anti-PTPRZ (Santa Cruz) antibodies recognize the HNK-1 epitope on sPTPRZ, and that CSF sPTPRZ has this epitope. We then expected that PTPRZ in glioma cells would have the HNK-1 epitope. Unexpectedly, however, PTPRZ was detected in LN-229 with antiphosphacan but not with Cat-315 or anti-PTPRZ (Santa Cruz) (Fig. 2*D*), indicating the absence of the HNK-1 epitope.

### Glycosylation analysis of PTPRZ in the xenograft model

**Figure 3:**
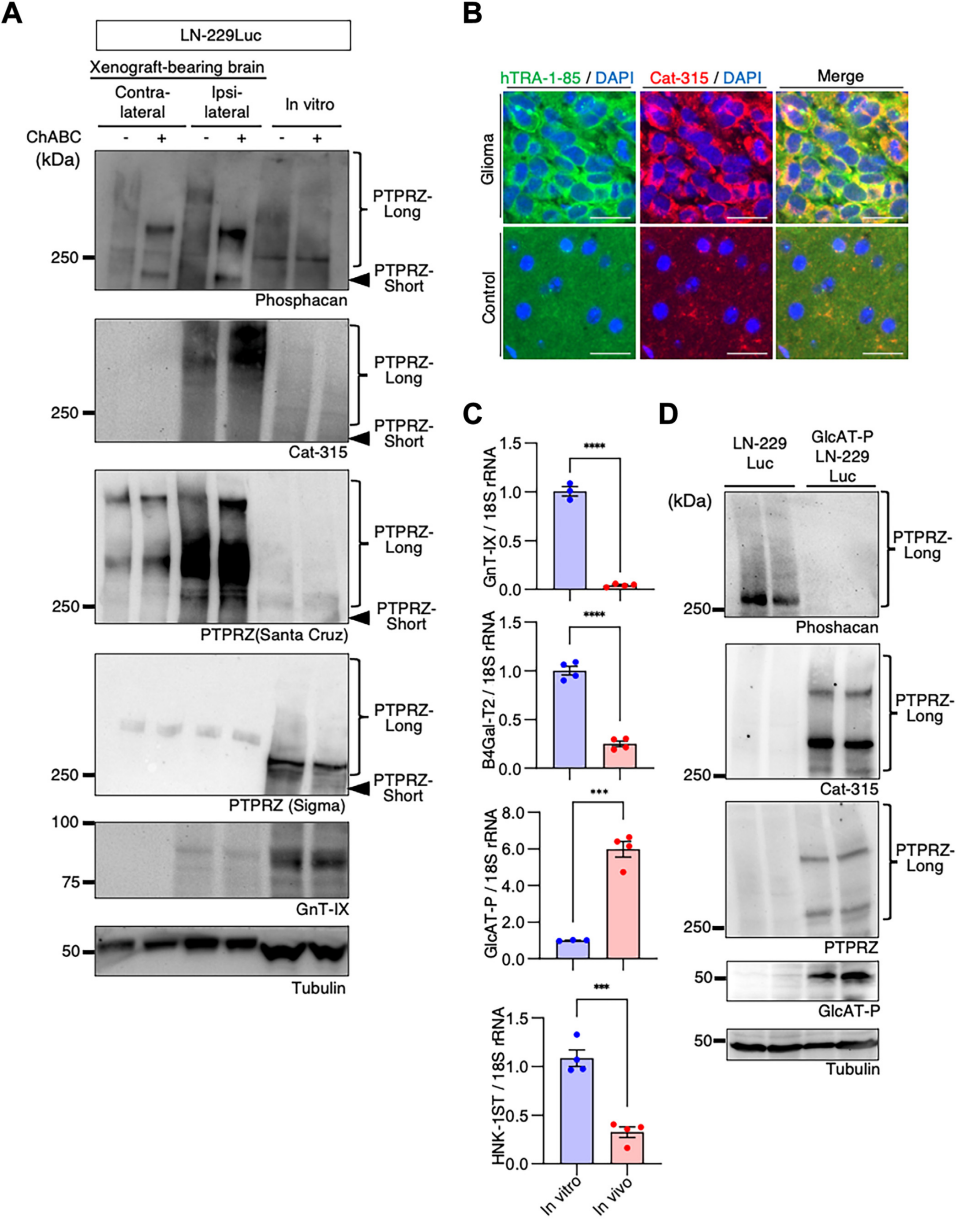
**Increased HNK-1 epitope on PTPRZ in glioma cells in mouse brains.***A*, the expression of PTPRZ and its glycosylated form was assessed by immunoblot analysis. Mouse brains xenografted with LN-229Luc were separated as ipsilateral and contralateral hemispheres and lysed. As a comparison, LN-229Luc lysates from cell culture (*in vitro*) were also prepared. The samples were treated with or without ChABC and probed with a series of antibodies: phosphacan, PTPRZ (Santa Cruz), Cat-315, PTPRZ (Sigma), and GnT-IX, with tubulin as the loading control. A typical immunoblot is shown. *B*, immunofluorescent images of glioblastoma xenograft-bearing brains and adjacent normal brains for hTRA-1 to 85 (*green*) and Cat-315 (*red*). Scale bar represents 20 μm. *C*, quantification of the transcripts of glycosyltransferases involved in the synthesis of the HNK-1-capped O-Man core M2 structure in LN229Luc *in vitro* (cell culture) and *in vivo* (FACS-sorted from brain xenografts with LN229Luc). *D*, immunoblot analyses of PTPRZ in cell lysates of LN-229Luc and ΔGnT-IX-LN-229Luc. A series of antibodies were used (phosphacan, PTPRZ [Santa Cruz], Cat-315, and GlcAT-P) with tubulin as the loading control. ChABC, chondroitinase ABC; FACS, fluorescence-activated cell sorting; GnT-IX, N-acetylglucosaminyltransferase IX; HNK-1, human natural killer-1; O-Man, O-mannosyl; PTPRZ, protein tyrosine phosphatase receptor Z.

Emerging reports indicate that extrinsic signals—such as growth factors and neuronal activity, and resulting transcriptional and chromatin remodeling—are necessary for astrocyte maturation; *in vitro* cultured astrocytes lack the expression of many mature astrocyte-specific genes (38, 39). Such extrinsic signals may also be critical to upregulate glycosylation enzyme genes for the addition of the HNK-1 epitope to PTPRZ in glioma. We therefore investigated whether PTPRZ was modified with the HNK-1 epitope in LN-229Luc transplanted into mouse brains. Unfortunately, Western blot analysis of fluorescence-activated cell sorting (FACS)-sorted LN-229Luc cells was unsuccessful because of severe proteolytic degradation. We therefore decided to analyze xenograft mouse brains without glioma separation. At 6 weeks after glioma injection, lysates from the contralateral cortex had endogenous PTPRZ signal detected using antiphosphacan and anti-PTPRZ (Santa Cruz), whereas lysates from the ipsilateral cortex had strong signal using anti-PTPRZ (Santa Cruz) and Cat-315; these findings indicate that the HNK-1 epitope was expressed in transplanted LN-229 cells. In contrast, these signals were absent in LN-229 lysates from cell culture (Fig. 3*A*). Moreover, LN-229 lysates had signal detected using anti-PTPRZ (Sigma), which recognizes a peptide portion specific to PTPRZ-long, whereas lysates from the ipsilateral cortex did not have positive signals. These results suggest that glioma PTPRZ in the xenograft model receives additional glycosylation, such as the HNK-1 epitope, which then hinders the epitopes for antiphosphacan and anti-PTPRZ (Sigma). The immunohistochemical analysis of tumor xenografts confirmed that Cat-315 signals were present in human glioma cells (detected with human-specific antigen TRA-1–85; Fig. 3*B*). We then expected that the expression of glycosylation enzymes for the synthesis of HNK-1 epitope would be suppressed in LN-229 cells cultured *in vitro*, whereas these expression levels would be upregulated in cells in the xenograft model, possibly by extrinsic signals *in vivo*. To explore this idea, we performed FACS on LN-229Luc cells from xenografted mouse brains and quantified the mRNA levels of a series of HNK-1-related glycosylation enzymes. Compared with the cells cultured *in vitro*, the mRNA levels of GnT-IX, β1,4-galactosyltransferase 2 (B4Gal-T2), and HNK-1ST were markedly lower in the *in vivo* sample (GnT-IX, 3.3%; B4Gal-T2, 25%; and HNK-1ST, 30%), whereas GlcAT-P mRNA levels in the cells *in vivo* were six times higher than those *in vitro* (Fig. 3*C*). These findings indicate that GlcAT-P expression in glioma cells is specifically upregulated by extrinsic signals in the brain. Indeed, ectopic GlcAT-P expression changed the glycan epitope of PTPRZ in LN-229 cells cultured *in vitro*; PTPRZ was detectable using anti-Cat-315 and -PTPRZ (Santa Cruz) antibodies, whereas reactivity with antiphosphacan disappeared (Fig. 3*D*). Notably, a higher molecular weight band—detected with anti-Cat-315 and -PTPRZ (Santa Cruz) antibodies—emerged with GlcAT-P expression, similar to that observed in brain lysates from xenograft glioma model mice (Fig. 3*A*).

### Knockdown of the O-Man branching enzyme GnT-IX reduces glioma growth

**Figure 4:**
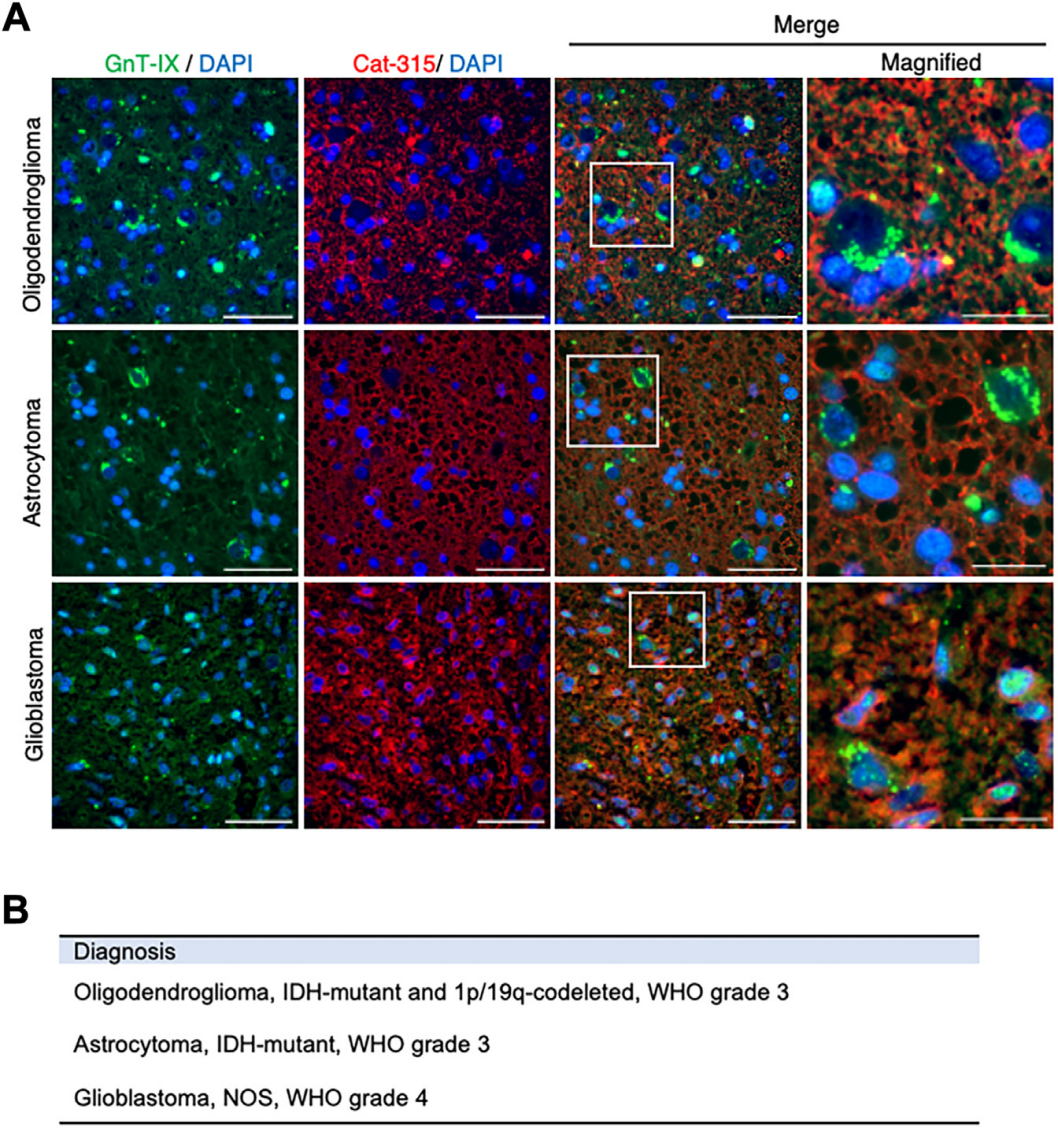
**GnT-IX expression in glioma tissues.***A*, immunofluorescent images of brain sections from patients with oligodendroglioma, astrocytoma, and glioblastoma. Sections were stained for GnT-IX (*green*), Cat-315 (*red*), and DAPI (*blue*). Scale bar represents 50 μm or 20 μm (magnified area). *B*, clinical information of the tumor specimens used in this study. DAPI, 4′,6-diamidino-2-phenylindole; GnT-IX, *N*-acetylglucosaminyltransferase IX.

**Figure 5:**
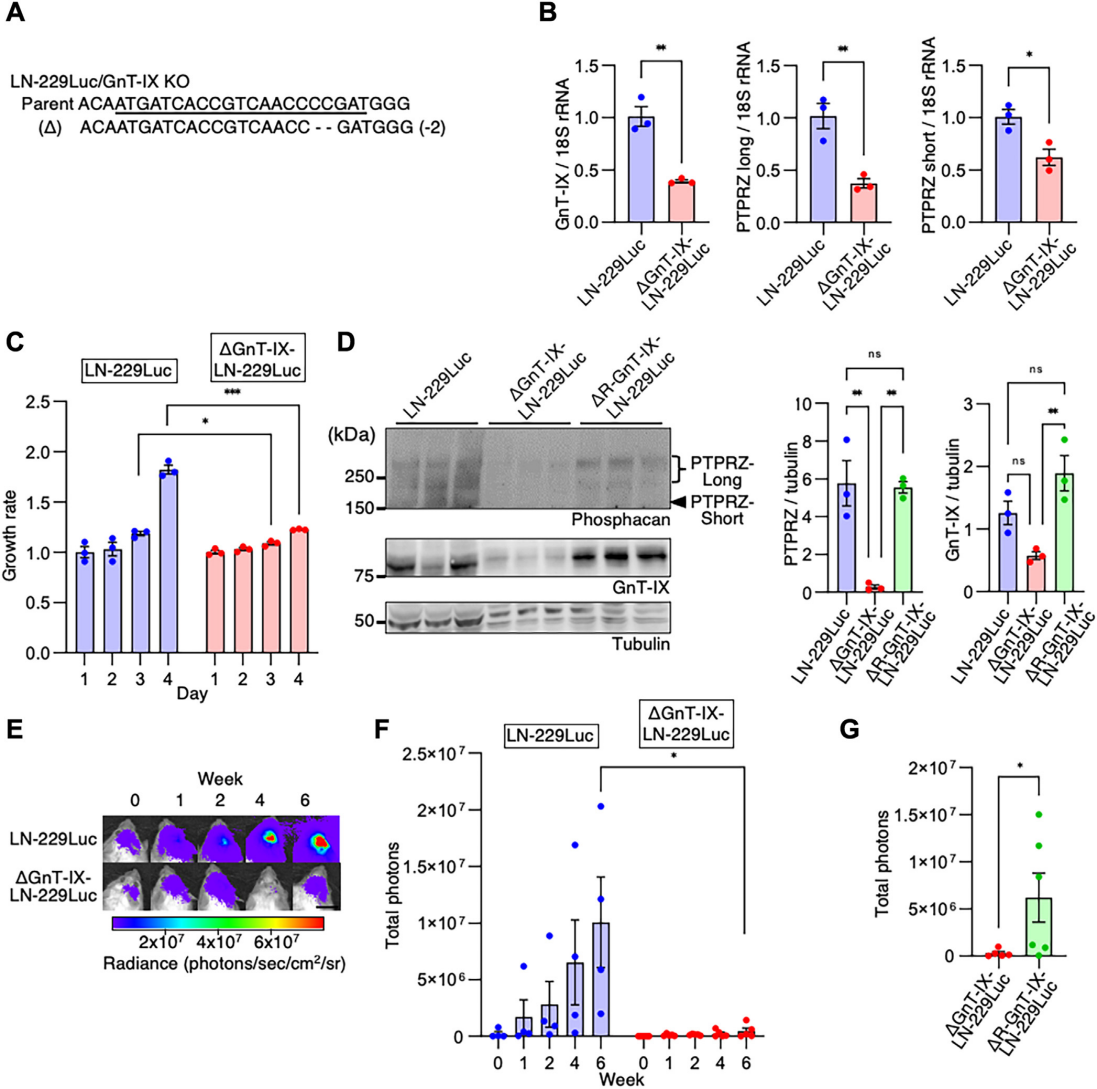
**GnT-IX depletion reduces PTPRZ protein levels and attenuates glioma growth both *in vitro* and *in vivo*.***A*, the targeted GnT-IX mutation sequences and the parental sequence in ΔGnT-IX-LN-229Luc cells are shown. The single-guide RNA target sequence is *underlined*. *B*, quantification of the PTPRZ-long and -short and GnT-IX mRNA transcripts, normalized to ribosomal RNA, in LN-229Luc and ΔGnT-IX-LN-229Luc cells. Data are shown as the mean ± SEM (n = 3). Student’s *t* test; ∗∗*p* < 0.01. *C*, cell growth rates of LN-229Luc and ΔGnT-IX-LN-229Luc cells with day 1 set as 1. Data are shown as the mean ± SEM (n = 3). Student’s *t* test; ∗*p* < 0.05, ∗∗∗*p* < 0.001. *D*, immunoblot analyses of PTPRZ and GnT-IX, with tubulin as the loading control, in the cell lysates of LN-229Luc, ΔGnT-IX-LN-229Luc, and ΔR-GnT-IX-LN-229Luc. Data are shown as the mean ± SEM (n = 3). Student’s *t* test; ∗*p* < 0.05 and ∗∗*p* < 0.01. *E*, *in vivo* bioluminescent analysis to monitor the intracranial tumor growth of glioblastoma xenografts derived from LN-229Luc and ΔGnT-IX-LN-229Luc in mice. Representative bioluminescent images on the indicated days are shown. Scale bar represents 1 cm. *F*, mean bioluminescence of the glioblastoma xenografts with LN-229Luc and ΔGnT-IX-LN-229Luc from 0 to 6 weeks. Data are shown as the mean ± SEM. n = 4 mice per group (LN-229Luc-derived xenografts) or n = 5 mice per group (ΔGnT-IX-LN-229Luc-derived xenografts). Student’s *t* test; ∗*p* < 0.05. *G*, mean bioluminescence of the glioblastoma xenografts with ΔGnT-IX-LN-229Luc and ΔR-GnT-IX-LN-229Luc at 6 weeks after transplantation. Data are shown as the mean ± SEM. n = 5 mice per group (ΔGnT-IX-LN-229Luc-derived xenografts) or n = 6 mice per group (ΔR-GnT-IX-LN-229Luc derived xenografts). One-tailed Mann–Whitney *U* test; ∗*p* < 0.05. GnT-IX, *N*-acetylglucosaminyltransferase IX; PTPRZ, protein tyrosine phosphatase receptor Z.

An important role of protein glycosylation is to protect carrier proteins (30). Furthermore, it has previously been reported that GnT-IX deficiency results in reductions of both the Cat-315 epitope and PTPRZ in mouse brains (28, 29). We therefore considered whether O-Man core M2 glycan (synthesized by GnT-IX) plays a role in maintaining PTPRZ levels in glioma cells. Because GnT-IX expression was markedly downregulated in LN-229 in xenograft mouse brains compared with those *in vitro* (Fig. 3*A*), we first examined GnT-IX expression in human glioma samples. We immunohistochemically analyzed a series of gliomas (oligodendroglioma, IDH mutant and 1p/19q-codeleted, WHO grade 3; astrocytoma, IDH mutant, WHO grade 3; and glioblastoma, mot otherwise specified, WHO grade 4) to verify the expression of GnT-IX and PTPRZ with the HNK-1 epitope (Fig. 4*A*). All three glioma types were Cat-315 positive, and the plasma membranes were especially strongly stained. Moreover, all glioma types had GnT-IX-signals in the perinuclear region. These findings indicate that PTPRZ and GnT-IX are simultaneously expressed in glioma cells. We then disrupted the GnT-IX gene in LN-229Luc cells using CRISPR genome-editing technology (Fig. 5*A*). Quantitative PCR analysis revealed that, in the resulting ΔGnT-IX-LN-229Luc (Fig. 5*B*), GnT-IX mRNA levels were reduced to about 50% of those of parental LN-229Luc cells. Moreover, the mRNA levels of PTPRZ-long and PTPRZ-short were also reduced in ΔGnT-IX-LN-229Luc cells; the mechanisms underlying these findings are unknown. The *in vitro* proliferation assay revealed that ΔGnT-IX-LN-229Luc cells had significantly reduced cell growth compared with LN-229Luc cells (Fig. 5*C*). To examine the off-target effects of CRISPR/CRISPR-associated protein 9 (Cas9)-mediated unintended cleavage and mutations at untargeted genomic sites in ΔGnT-IX-LN-229Luc cells, we constructed ΔR-GnT-IX-LN-229Luc cells in which GnT-IX was expressed ectopically. Western blot analysis revealed that not only GnT-IX levels but also PTPRZ levels were significantly reduced in ΔGnT-IX-LN-229Luc cells, whereas both GnT-IX and PTPRZ expression was restored in ΔR-GnT-IX-LN-229Luc cells (Fig. 5*D*). This result clearly indicates that GnT-IX is critical for maintaining cellular PTPRZ levels. We therefore expected that GnT-IX knockdown would result in retarded tumor growth in a xenograft glioma model. Indeed, mice transplanted with ΔGnT-IX-LN-229Luc had significantly reduced tumor growth compared with those transplanted with LN-229Luc (Fig. 5, *E* and *F*), whereas ΔR-GnT-IX-LN-229Luc transplantation recovered tumor growth in the xenograft glioma model (Fig. 5*G*). Although xenograft tumor growth was markedly decreased by GnT-IX knockdown, the immunohistochemical analysis of residual tumor sections derived from ΔGnT-IX-LN-229Luc revealed Cat-315 signals in glioma cells (Fig. S2). However, it remains unclear whether PTPRZ expression is essential for glioma growth, or whether the inhibition of tumor growth by GnT-IX deletion is also mediated by the impaired function of glycoproteins other than PTPRZ. Finally, we purified PTPRZ from the cell lysates of LN-229Luc and ΔGnT-IX-LN-229Luc cultured *in vitro* and treated with trypsin and Lys-C proteases and used this purified PTPRZ for MS–MS analysis. The LN-229Luc cells had O-Man core M2 glycan attached to Thr93 of PTPRZ-derived peptides. Moreover, in the ΔGnT-IX-LN-229Luc cells, one HexNAc residue was missing in the corresponding glycopeptide (Fig. S3), indicating that O-Man core M2 glycan in PTPRZ is synthesized by GnT-IX. Collectively, these findings indicate that GnT-IX inhibition may be a novel therapeutic strategy for glioma.

## Discussion

Gliomas are invasive malignant tumors with indistinct boundaries, and their growth disrupts brain function, induces seizures, and ultimately leads to death. While the development and improvement of local therapies, such as surgery and radiotherapy, are important for the local control of gliomas, they appear to be unable to cure the disease because of its highly invasive nature. New and effective chemotherapeutic agents are therefore urgently needed; for their successful development, we first need to identify therapeutic target molecules for gliomas.

Similar to the current study, several groups have shown that targeting PTPRZ effectively attenuates tumor growth in xenograft models (19, 20, 31). Because PTPRZ-deficient mice are healthy, fertile, and have apparently normal gross anatomy, the inhibition of PTPRZ to treat glioma may be free of mechanism-based toxicity. Nevertheless, because of its relatively large catalytic pocket, it is challenging to develop specific PTPRZ inhibitors that can cross the blood–brain barrier. The present study shows for the first time that targeting PTPRZ glycosylation is a promising therapeutic strategy. We found that O-Man core M2 glycosylation plays a role in maintaining cellular PTPRZ levels because GnT-IX knockdown reduced PTPRZ levels in glioma cells and led to significantly reduced tumor growth in a xenograft glioma model. The way in which GnT-IX deficiency reduces cellular PTPRZ levels has yet to be clarified. GnT-IX was originally identified as a homolog of the N-glycan branching enzyme GnT-V; however, GnT-V is ubiquitously expressed, whereas GnT-IX exhibits brain-specific expression (40) that is epigenetically regulated (41, 42). Notably, GnT-IX mRNA levels in glioma cells cultured *in vitro* were almost 30 times higher than those in xenograft mouse brains, suggesting that the epigenetic inhibition of GnT-IX expression is lost in cells cultured *in vitro.* These relatively high levels of GnT-IX expression in cell culture will be beneficial for screening GnT-IX inhibitors as potential glioma therapeutics. Furthermore, although GnT-IX knockdown leads to significantly reduced tumor growth, Cat-315 signals were clearly detected in residual tumor sections transplanted with ΔGnT-IX-LN-229Luc cells, indicating that PTPRZ is expressed in glioma cells in the xenograft model. However, it has yet to be clarified whether this is because PTPRZ expression is essential for glioma growth or because glycoproteins other than PTPRZ are also GnT-IX substrates and play a critical role for glioma growth. The current study revealed the presence of O-Man core M2 glycan synthesized by GnT-IX in PTPRZ. To date, the MS–MS analysis of O-Man glycans derived from mouse brain PTPRZ has revealed marked heterogeneity, such as Lewis^x^ epitopes, terminally sialylated glycans, sulfated and sialylated glycans, sulfated N-acetyllactosamine, and HNK-1 epitopes (22). In normal mouse brains, the majority of O-Man glycans are sialylated, and HNK-1-capped O-Man glycans are rare. In contrast, glioma cells express increased levels of HNK-1-capped O-Man glycans on PTPRZ; this is crucial for recognition by Cat-315 and anti-PTPRZ (Santa Cruz) antibodies. HNK-1 capped O-Man core M2 glycans are present in PTPRZ only, whereas the HNK-1 epitope can be attached to other types of glycans: N-glycans in the α-amino-3-hydroxy-5-methyl-4-isoxazolepropionic acid receptor subunit GluA2, and on a novel type of glycans in aggrecans. Genetic deletion of either B4Gal-T2, GlcAT-P, or HNK-1ST results in the significant loss of these HNK-1 epitopes, and mice deficient in these enzymes show impaired spatial learning/memory and motor coordination/learning (43, 44). These findings indicate the functional importance of the HNK-1 epitope for maintaining normal brain function and suggest that the inhibition of enzymes that are important for the synthesis of the HNK-1 epitope may have cognitive side effects. However, these abnormalities are not observed in GnT-IX-deficient mice, which have attenuated astrogliosis and enhanced remyelination in a demyelination model (29). Although physiological substrates of GnT-IX other than PTPRZ have yet to be determined, we consider that targeting GnT-IX may inhibit tumor growth without serious side effects.

From a therapeutic viewpoint, GnT-IX inhibitors should cross the blood–brain barrier and act in Golgi apparatus; the development of such inhibitors may therefore be challenging. Nevertheless, there are some existing examples of glycosylation enzyme inhibitors. It has been reported that fluorosamine—a fluorinated analog of GlcNAc that cannot be converted to GalNAc by 4-epimerase, thus resulting in GalNAc depletion *in vivo*—reduces chondroitin sulfate levels and leads to the promotion of remyelination (45). Moreover, several kinds of small-molecule glucosylceramide synthase inhibitors that can penetrate the CNS are in clinical development (46). Alternatively, an antibody specific to PTPRZ with HNK-1-capped glycans may be a unique therapeutic agent because blood–brain barrier leakage is often observed around high-grade glioma regions. As demonstrated in the present study, a deeper understanding of tumor-specific glycosylation may provide novel potential therapeutic strategies.

## Experimental procedures

### Ethics statement

Animal studies were approved by the Animal Experiments Committee of Fukushima Medical University and the Institutional Animal Care and Use Committee of the Institute of Medical Science, The University of Tokyo, in compliance with their respective animal experiment guidelines. The clinical study was approved by the ethical committee of Fukushima Medical University (approval no.: 29378).

### Human samples

CSF samples and tumor specimens from patients with gliomas were collected by craniotomy at Fukushima Medical University from 2016 to 2022. Samples were pathologically diagnosed based on WHO 2021 criteria. Clinical information of patients who provided CSF samples and tumor specimens is shown in Table S1.

### Materials

Unless otherwise noted, materials were obtained as follows: TriPure Isolation Reagent and Transcriptor First Strand complementary DNA (cDNA) Synthesis Kit from Roche; tissue culture medium and reagents, including Dulbecco’s modified Eagle’s medium (DMEM), from Invitrogen; protein molecular weight standards from Bio-Rad; bicinchoninic acid protein assay reagents from Thermo Fisher Scientific; and all other chemicals from Sigma or FUJIFILM Wako Chemicals. Antiphosphacan antibody was prepared against recombinant rat phosphacan (34). The following antibodies were purchased: anti-human TRA-1–85/CD147 (catalog no.: MAB3195; R&D Systems) and antitubulin (catalog no.: T5168, clone#B-5-1-2; Sigma) mouse immunoglobulin G (IgG); anti-PTPRζ (catalog no.: sc-33664; Santa Cruz Biotechnology, referred to as “anti-PTPRZ [Santa Cruz]”) and Cat-315 (catalog no.: MAB1581; Merck Millipore) mouse immunoglobulin M (IgM); and anti-PTPRZ1 (catalog no.: HPA015103; Sigma, referred to as “anti-PTPRZ1 [Sigma]”), anti-GnT-IX (catalog no.: 16993-1-AP; ProteinTech), anti-3-beta-glucuronosyltransferase 1 (GlcAT-P; catalog no.: ab199156; abcam), and anti-histone H3 (catalog no.: H0164; Sigma–Aldrich) rabbit IgG. The following horseradish peroxidase–conjugated secondary antibodies were used: goat antimouse IgM (catalog no.: SAB-110; StressGen), goat anti-rabbit IgG (catalog no.: NA934; GE Healthcare), and goat antimouse IgG (catalog no.: NA931-1ML; Cytiva). The following fluorescently labeled secondary antibodies were used: Alexa Fluor 546 goat antimouse IgM (catalog no.: A21045; Life Technologies), Alexa Fluor 546 goat anti-rabbit IgG (catalog no.: A11010; Invitrogen), Alexa Fluor 488 goat antimouse IgG (catalog no.: A-11001; Molecular Probes), and Alexa Fluor 488 goat anti-rabbit IgG (catalog no.: A-11008; Molecular Probes).

### Cell culture

HEK293T cells (purchased from the American Type Culture Collection) were maintained in DMEM containing 10% fetal calf serum. Human glioblastoma LN-229 cells (catalog no.: CRL-2611; American Type Culture Collection) were maintained in DMEM (catalog no.: D5796; Sigma) supplemented with 10% fetal bovine serum (Equitech-Bio, Inc) and penicillin–streptomycin solution (catalog no.: 168-23191; FUJIFILM).

### Plasmids

All PCR steps were performed using Tks Gflex DNA Polymerase (TaKaRa). Human PTPRZ-long (6948 bp) was cloned by real-time PCR using total RNA from LN-229 cells with the primers 5′-GGAATTCGATATCAAATGCGAATCCTAAAGCGTTT-3′ (forward) and 5′-ACGGTATCGATAAGCTTTAAACTAAAGACTCTAAG-3′ (reverse) and subcloned into the EcoRV site of the pBluescript II SK vector (Stratagene) using an In-Fusion HD Cloning Kit (TaKaRa). For C-terminal sPTPRZ754-His, extracellular regions of PTPRZ-long after the signal peptide were amplified using PCR with the primers 5′-CCGGCCAGATCTCCCTACTACAGACAACAGAGAAA-3′ (forward) and 5′-CGTCGACCTGCAGCCTTAGTGATGGTGGTGATGATGGTGGTGATGATGATTGTATACCGGTTGGGT-3′ (reverse) and inserted between the SmaI and SacII sites of pDisplay (Thermo Fisher Scientific). For retroviral GnT-IX or GlcAT-P expression, rat GnT-IX cDNA was amplified using the primers 5′-AGTGTGGTGGTACGGGAATTCATGATCACCGTCAACCCCGA-3′ (forward) and 5′-ACCGGCGCTCAGCTGGAATTCTCACAGACAGCCCTGGCACA-3′ (reverse), or rat GlcAT-P cDNA was amplified using the primers 5′-AGTGTGGTGGTACGGGAATTCATGGGTAATGAGGAGCTGTG-3′ (forward) and 5′-ACCGGCGCTCAGCTGGAATTCTCAGATCTCCACCGAGGGGT -3′ (reverse); these were then inserted into the EcoRI site of pMX-neo or pMX-puro, respectively.

### Establishment of LN-229Luc, ΔPTPRZ-LN-229Luc, ΔGnT-IX-LN-229Luc, and ΔR-GnT-IX-LN-229Luc cells

For both the *in vivo* monitoring of tumor growth and FACS, LN-229 cells were labeled with luciferase and GFP (LN-229Luc) using RediFect Lentiviral particle (Red-FLuc-GFP; PerkinElmer). At 48 h after infection, GFP-positive cells underwent FACS (BD FACS Melody Cell Sorter; BD Biosciences).

LN-229Luc cells with PTPRZ or GnT-IX knockdown were generated using the CRISPR–Cas9 system. Sense and antisense oligonucleotides were designed for insertion into the BbiI site of the pX458 (for ΔGnT-IX-LN-229Luc) or pX459 (for ΔPTPRZLN-229Luc) bicistronic expression vector expressing Cas9 and synthetic single-guide RNA (Addgene) as follows: 5′-CACCGATGGTATCATAAACGACTCGAGG-3′ and 5′-AAAC CGAGTCGTTTATGATACCATC-3′ for PTPRZ, and 5′-CACCGATGGTATCATAAACGACTCGAGG-3′ and 5′-AAACCGAGTCGTTTATGATACCATC-3′ for GnT-IX. These were used to produce px459-PTPRZ and px458-GnT-IX, respectively (47). The DNA oligonucleotides were annealed and incorporated into pX458 or px459 vectors linearized with BbiI restriction enzyme. Next, px459-PTPRZ or pX458-GnT-IX was introduced into LN-229 cells using an NEPA21 electroporator (NEPA GENE). After transfection, cells were treated with 3 mg/ml puromycin for 24 h (for px459-PTPRZ), or GFP+ cells were collected using a BD FACS Melody Cell Sorter (for px458-GnT-IX), and the cells were replated. Genomic DNA was purified from cells using a GeneJET Genomic DNA Purification Kit (Thermo Fisher Scientific), and mutation was verified by sequencing. Plat-GP cells, a 293T-derived murine leukemia virus–based packaging cell line, were cotransfected with pMXs-GnT-IX-neo and pMDG encoding vesicular stomatitis virus envelope protein G using Lipofectamine 2000 (Invitrogen) according to the manufacturer’s instructions. Supernatants were harvested 48 h after transfection. The ΔGnT-IX-LN-229Luc cells were then transduced with retrovirus-containing supernatants of transfected Plat-GP cells and selected using 2 mg/ml neomycin. The resistant cells, ΔR-GnT-IX-LN-229Luc, were used for all further experiments.

### Isolation of PTPRZ and glycopeptide preparation

LN-229Luc and ΔGnT-IX-LN-229Luc cells were lysed with TPER Tissue Protein Extraction Reagent (Thermo Fisher Scientific) containing a complete protease inhibitor cocktail (EDTA free; Nacalai Tesque). PTPRZ was immunoaffinity purified from the resulting cell lysates (2 mg protein) using antiphosphacan antibody bound to Dynabeads protein G (Life Technologies). The beads were washed with wash buffer, rinsed five times with PBS and once with water, and subsequently boiled with 40 μl phase transfer surfactant (12 mM sodium deoxycholate and 12 mM sodium *N*-lauroylsarcosinate in 50 mM Hepes [pH 8.0]) (48) to elute the proteins. Proteins were then reduced, alkylated, and digested using 250 ng Trypsin/Lys-C mix (Mass Spectrometry Grade; Promega) at 37 °C for 16 h. O-glycopeptides were enriched by acetone precipitation (49).

### LC–MS/MS analysis

Glycopeptides were resuspended in 2% acetonitrile solution with 0.1% trifluoroacetic acid and analyzed using an Orbitrap Fusion Lumos Tribrid mass spectrometer (Thermo Fisher Scientific) coupled with a Vanquish Neo UHPLC system (Thermo Fisher Scientific). Glycopeptides were first trapped using a precolumn (C18 Acclaim PepMap 100 C18 Trap Cartridge; Thermo Fisher Scientific) and then separated using an analytical column (Aurora UHPLC Column [C18, 0.075 × 250 mm, 1.7 μm particle size, IonOpticks]). Elution was performed with a linear gradient of 2% to 30% solvent B over 55 min at a flow rate of 200 nl/min (solvent A: 0.1% formic acid in water and solvent B: 0.1% formic acid in acetonitrile). The mass spectrometer was operated in data-dependent acquisition mode. The MS parameters were as follows: spray voltage, 2.0 kV; capillary temperature, 275 °C; S-lens RF level, 30; scan type, full MS; scan range, *m*/*z* 350 to 1500; resolution, 120,000; polarity, positive; automatic gain control target, standard; and maximum injection time, auto. The MS/MS parameters were as follows: activation type, higher-energy C-trap dissociation; resolution, 15,000; automatic gain control target, standard; maximum injection time, auto; higher-energy C-trap dissociation collision energy, 28%; dynamic exclusion, 60 s; loop time, 3 s; and isolation window, 2 *m*/*z*.

Acquired raw data were processed using Byonic software, version 4.3.4 (Protein Metrics). Precursor mass tolerance was set to 10 ppm, and fragment mass tolerance was set to 0.02 Da. The maximum number of missed cleavages was set to two. Methionine oxidation and cysteine carbamidomethylation were included as dynamic modifications.

### Cell proliferation assay

Cell Counting Kit-8 (Dojindo) was used for the cell proliferation assay; 10,000 cells were plated per well, and proliferating cell viability was determined at 1, 2, 3, and 4 days after incubation.

### Western blot analysis

Either sPTPRZ754 in media from HEK293T cells and CSF samples from glioma patients (10 μl) or lysates of xenograft mouse brains (20 μg protein) were digested with 0.2 mU ChABC (Sigma) in Tris-acetate buffer (pH 7.4) containing a protease inhibitor cocktail (Nacalai) for 1 h at 37 °C. Parts of the CSF samples were additionally digested with 3 μl endo-β-galactosidase (R&D Systems) in sodium citrate buffer (pH 6.0) for 1 h at 37 °C with or without 2 μl sialidase (*Arthrobacter ureafaciens* neuraminidase) treatment and PNGase F (New England BioLabs). The samples underwent sodium dodecyl sulfate–polyacrylamide gel electrophoresis (3–10% gradient gels; Atto) and were transferred to nitrocellulose membranes. After blocking with 5% nonfat dried milk in PBS (pH 7.4) containing 0.1% Tween-20 for 30 min, the membranes were probed with antiphosphacan (1:1000 dilution), anti-PTPRZ (Santa Cruz; 1:1000 dilution), Cat-315 (1:1000 dilution), anti-PTPRZ1 (Sigma; 1:750 dilution), anti-GnT-IX (1:1000 dilution), anti-tubulin (1:1000 dilution), and anti-histone H3 (1:1000 dilution) antibodies for 2 h, and with the appropriate horseradish peroxidase–conjugated secondary antibodies (1:3000 dilution) for 1 h. The blots were developed using Western Lightning Ultra (PerkinElmer) for PTPRZ and Cat-315 detection and Western Lightning ECL Pro (PerkinElmer) for all other antibodies. Signals were detected with the ChemiDoc Touch MP (Bio-Rad) and quantified using Image Lab Software (Bio-Rad).

### Immunohistochemistry and histology

Glioma paraffin sections (5 μm thickness) were obtained from the Fukushima Medical University Hospital. Mouse brains were transcardially perfused with PBS and 0.1 M phosphate-buffered 4% paraformaldehyde before being paraffin embedded and sliced into 4 μm-thick sections. The sections were deparaffinized in xylene for 10 min, rehydrated in an ethanol series (100%, 95%, and 70%), and incubated with HistoVT One (catalog no.: 06380-05; Nacalai Tesque) for 20 min at 90 °C for antigen retrieval and with Blocking One Histo (catalog no.: 06349-64; Nacalai Tesque) for 10 min at 25 °C. Next, sections were incubated with primary antibodies (Cat-315, 1:250 dilution; anti-MGAT5b, 1:200 dilution; anti-human TRA-1-85/CD147, 1:50 dilution) for 1 h at 25 °C and with fluorescently labeled secondary antibodies (1:200 dilution) for 45 min at 25 °C. After washing, ProLong Glass Antifade Mountant with NucBlue (catalog no.: P36985; Invitrogen) was applied to the sections. Images were captured using a fluorescent microscope (catalog no.: BZ-X800; Keyence).

### Human PTPRZ expression

HEK293T cells were transfected with pDisplay-hPTPRZ754-His with or without GlcAT-P-HNK-1ST (50) and GnT-IX-pcDNA (40) using PEI Max and were then cultured for 48 h with protease inhibitor cocktail (1:200 dilution, catalog no.: P8349; Sigma). sPTPRZ754-His was pulled down from culture media using Ni-Sepharose High Performance (Cytiva) and used for Western blot analysis.

### Real-time PCR analysis

The LN-229Luc cells cultured *in vitro* or isolated from mouse brains were used to isolate total RNA using a High Pure RNA Isolation Kit (Roche). RNA samples (1–5 μg) were then reverse-transcribed with random hexamers using a Transcriptor First-Strand cDNA Synthesis Kit (Roche) as per the manufacturer’s protocol. The amount of cDNA of specific genes was then quantified using a TaKaRa qPCR probe (TaKaRa) or the Universal ProbeLibrary (Roche) with TaqMan Master (Roche) and a LightCycler 96 system (Roche) in accordance with the manufacturers’ instructions. The primer and probe sequences are shown in Table S2. The relative expression of each gene was calculated using the comparative cycle threshold (2^−ΔΔCt^) method (51).

### Glioblastoma mouse model

SCID-Beige mice (females, 6–8 weeks old) were purchased from Charles River Japan. All surgeries and measurements were performed under i.p. injection of medetomidine (0.75 mg/kg)/midazolam (4 mg/kg)/butorphanol (5 mg/kg) anesthesia, and all efforts were made to minimize suffering. To establish a mouse model of glioblastoma for *in vivo* studies, LN-229Luc, ΔPTPRZ-LN-229Luc, ΔGnT-IX-LN-229Luc, and ΔR-GnT-IX-LN-229Luc cells were intracranially transplanted into the brains of SCID-Beige mice.

Briefly, 200,000 cells suspended in 2 μl RPMI1640 with l-glutamine (FUJIFILM Wako Chemicals) were transplanted using a 1702 RN Neuros Syringe (catalog no.: 65460-10; Hamilton) at a rate of 1.0 μl/min into the right cerebral hemisphere at a depth of 4 mm and 3 mm to the right of bregma. After being injected, the mice were kept warm and given an i.p. injection of atipamezole (0.75 mg/kg, Antisedan; Nippon Zenyaku Kogyo Co, Ltd) to wake them from the anesthesia. Bioluminescent imaging was performed 15 min after the i.p. injection of 200 μl d-luciferin (30 mg/ml in PBS; catalog no.: 14682, Cayman Chemical) using the IVIS Lumina II *In Vivo* Imaging system (PerkinElmer) with an exposure time of 1 min. Data analysis was performed using Living Image, version 4.0 (Caliper Life Sciences). For the biochemical analysis, 6 weeks after glioma injection, the brain cortex was divided at the midline and both hemicortices were lysed with T-PER Tissue Extraction Reagent (Thermo Fisher Scientific) containing a protease inhibitor cocktail.

### Isolation of glioma cells from glioblastoma-bearing mice

At 7 weeks after glioblastoma transplantation, mouse brains were dissected, mechanically disaggregated with scissors, and dissociated using an Adult Brain/Lung Dissociation Kit (Miltenyi Biotech) and gentleMACS Dissociator with Heater (Miltenyi Biotech) in accordance with the established protocol. Dissociated cells were suspended in PBS containing 0.5% bovine serum albumin, and GFP^+^ glioma cells were analyzed/sorted using BD FACS Aria II (BD Biosciences) with BD FACSDiva Software, version 8.0.2 (BD Biosciences).

### Statistical analysis

Data are presented as the mean ± SEM. All groups were tested for normality using the Shapiro–Wilk test, and outliers were detected with the Smirnov–Grubbs test. Comparisons between two groups were performed using the Student’s *t* test or Mann–Whitney *U* test. Multiple comparisons were performed by one-way analysis of variance with the Tukey–Kramer test. All analyses were performed using GraphPad Prism 9.1.2. (Statcon).

## Data availability

This study includes no data deposited in external repositories.

## Supporting information

This article contains supporting information.

## Conflict of interest

The authors declare that they have no conflicts of interest with the contents of this article.
