# Supplementary material for: Brain-specific glycosylation enzyme GnT-IX maintains levels of protein tyrosine phosphatase receptor PTPRZ, thereby mediating glioma growth
Source: J Biol Chem. 2023 Aug 4;299(9):105128. doi: 10.1016/j.jbc.2023.105128 (PMC10480537; doi:10.1016/j.jbc.2023.105128)
Supplement: Supporting Figures S1–S3 and Tables S1 and S2 [file mmc1.docx]

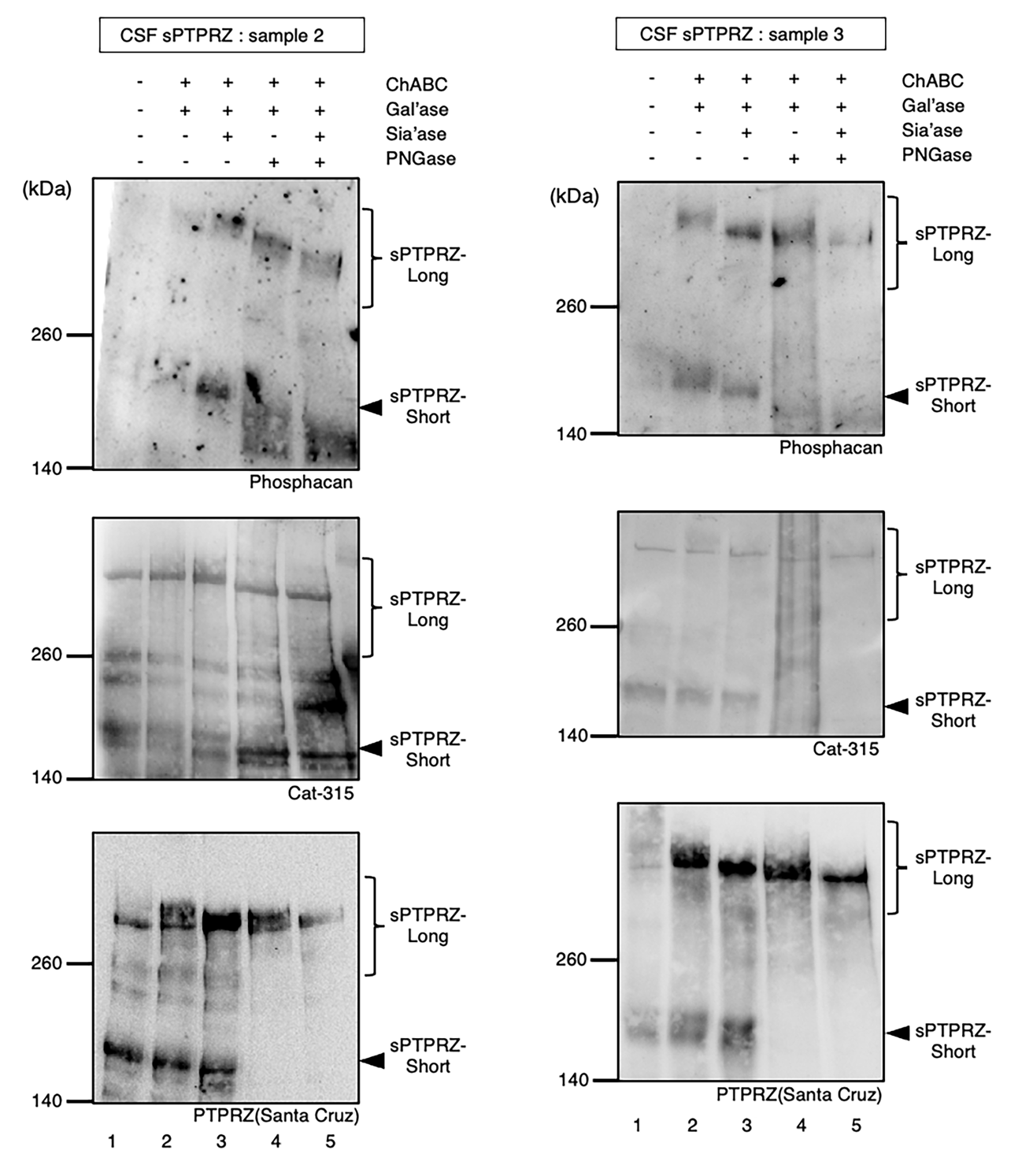


**Figure S1.**

Immunoblot analysis of sPTPRZ in the CSF of glioma patients. CSF samples from glioma patients (Sample 2 from glioblastoma, NOS, WHO grade 4, Sample 3 from oligodendroglioma, IDH-mutant and 1p/19q-codeleted, WHO grade3) were treated with or without chondroitinase ABC (ChABC), endo-β-galactosidase (Gal’ase), sialidase (Sia’ase), and peptide-N-Glycosidase (PNGase) before being probed with a series of antibodies: phosphacan, PTPRZ (Santa Cruz), and Cat-315.

**Figure S2.**

Immunofluorescent images of glioblastoma xenograft-bearing brains transplanted with LN-229Luc or ΔGnT-IX-LN-229Luc cells, with staining for hTRA-1-85 (green) and Cat-315 (red). Scale bars: 20 µm.


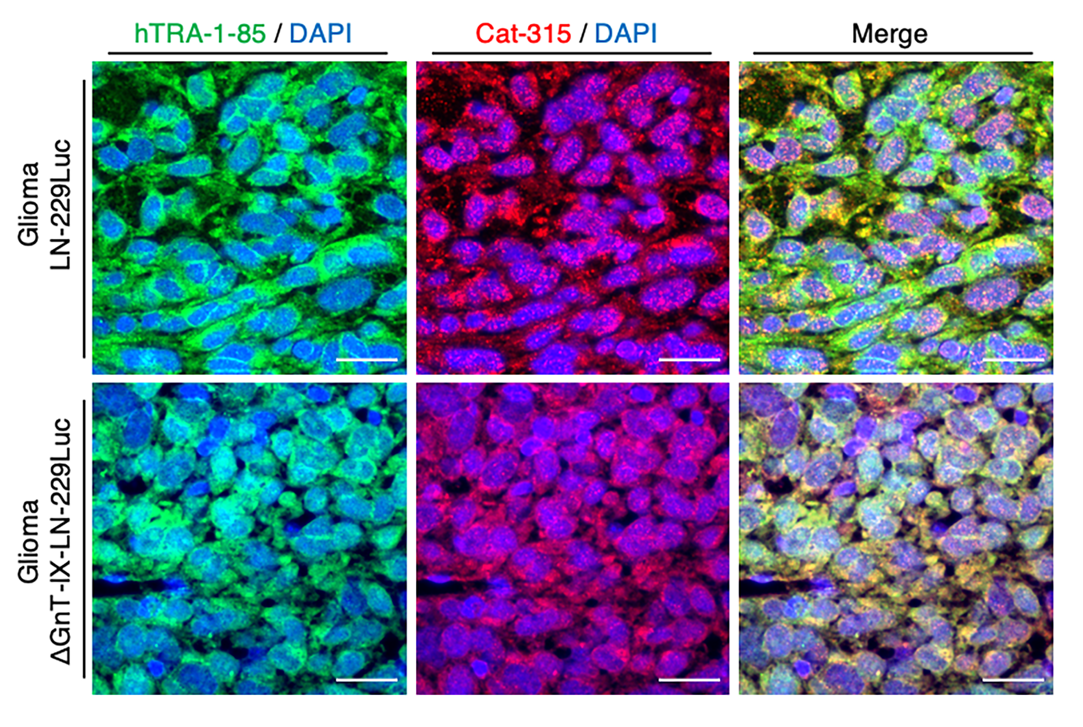


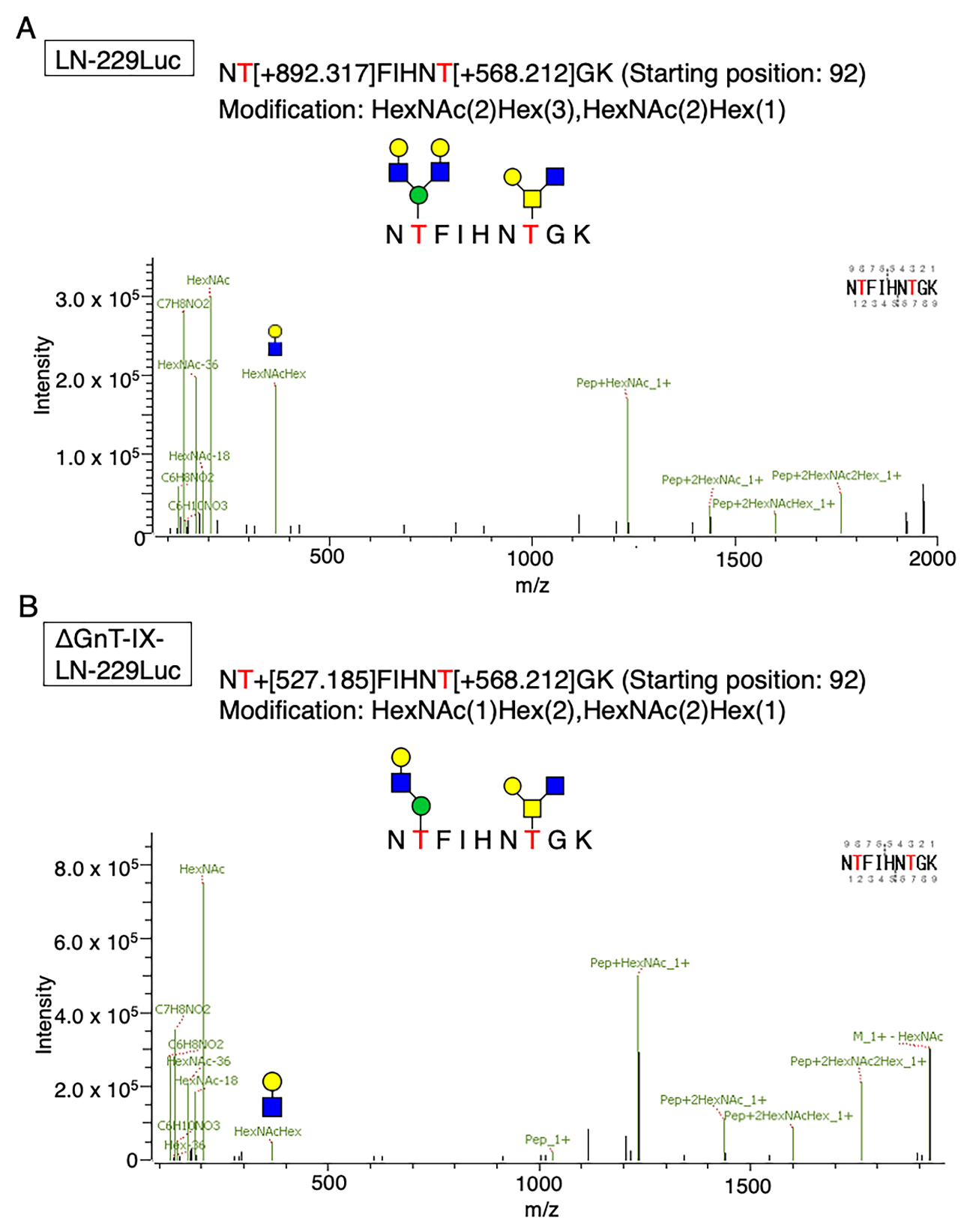


**Figure S3.**

Representative product ion spectra of PTPRZ O-glycopeptide. (A) aa92-aa100 arising from the precursor ion at 1246.532 (*z* = 2), prepared from LN-229Luc cells. (B) aa92-aa100 arising from the precursor ion at 1063.966 (*z* = 2), prepared from ΔGnT-IX-LN-229Luc cells.

**Supplementary Tables**

**Table S1**

**Clinical information of the CSF samples used in this study**

| **Sample** | **Diagnosis** | **Figure** |
| --- | --- | --- |
| 1 | Glioblastoma, IDH-wild type, WHO grade 4 | Fig.2 B |
| 2 | Glioblastoma, NOS, WHO grade 4 | Figure S1 |
| 3 | Oligodendroglioma, IDH-mutant and 1p/19q-codeleted, WHO grade 3 | Figure S1 |

**Table S2**

**Sequences of qPCR primers and probes used in this study**

| **Targeting gene**  **(Protein)** | **Primer and probe sequence (5’-3’)** |
| --- | --- |
| *PTPRZ1* | F: GACTCAGAAATAACTCCTGGATTCC |
| (PTPRZlong) | R: GACCAATACGAGACTCATGGCTA |
|  | FAM-CCTCTGCCTCTGAAACGTGGAACACTTCTG-BHQ-1 |
| *PTPRZ1* | F: TCCTCCAGACAACAGGATTTGG |
| (PTPRZshort) | R: TGGCTACTATTACTGGCCTCATTG |
|  | FAM- ACGGTCAACGTGGTATACTCGCAGACAACC-BHQ-1 |
| *MGAT5B* | F: ACCCTACGAGTACACCTGCG |
| (GnT-IX) | R: GGTAGGGCAGGGTCTGGAG |
|  | FAM-CACGCCTACATCCAGCACCAGGACTTCT-BHQ-1 |
| *B4GALT2* | F: CGTCTATGTCATCAACCAGCATG |
| (B4Gal-T2) | R: CCAGGTCCACATCGCTGAAG |
|  | FAM-AGCAGTCATAGGCGGCATCCTCCTTCA-BHQ-1 |
| *B3GAT1* | F: AGACGGGACATCCTAGCGATC |
| (GlcAT-P) | R: GGGTCACTGCCCTCATCCTTA |
|  | FAM-ATCGTGCTGCCCTGGACTCTGCTCAT-BHQ-1 |
| *CHST10* | F: AGACCCAGATGTGTACAGTGC |
| (HNK-1ST) | R: GTTGGCTTCAGTTCCTCAGGA |
|  | FAM-CTTCTCTTCTGGCAACTTCCTCACTTCCG-BHQ-1 |
| *18S ribosomal RNA* | F: GCAATTATTCCCCATGAACG |
|  | R: GGGACTTAATCAACGCAAGC |
|  | ProbeLibrary probe 48 (Roche) |

The probes for PTPRZ-long (PTPRZ1 isoforms 1, 4, and 5), PTPRZ-short (PTPRZ1 isoforms 2 and 3) and a series of glycosyltransferase genes were labeled with the fluorescent reporter dye FAM at its 5ʹ end and the quencher dye BHQ-1 at its 3ʹ end. The probe for ribosomal RNA was labeled with VIC at its 5ʹ end and the quencher dye BHQ-1 at its 3ʹ end.
